# A rare case of stent-assisted coil embolization of coronary artery aneurysm in the left main trigeminal position: Case report

**DOI:** 10.1097/MD.0000000000018173

**Published:** 2019-12-16

**Authors:** Yulin Chen, Minjie Liu, Kaihan Ren, Mingwei Wang, Fang Zhang, Dongyu Jia

**Affiliations:** a Department of Cardiology; b Department of Ultrasound, Hangzhou Normal University, Hangzhou, Zhejiang, China; c Department of Biology, Georgia Southern University, Statesboro, GA.

**Keywords:** coil embolization, coronary artery aneurysm, drug-eluting stent

## Abstract

**Rationale:**

Coronary artery aneurysms (CAAs) are uncommon in patients with acute coronary syndrome (ACS). We describe the clinical features and outcomes of stent-assisted coil embolization of a CAA in the trigeminal position.

**Patient concerns:**

We present a 73-year-old woman with a history of paroxysmal episodes of precordial pain since 1 year. Coronary computed tomography angiography (CTA) revealed an aneurysm (diameter: 9 mm) at the junction of the distal left main coronary artery and the anterior descending branch. Troponin I, CK-MB, creatinine and routine blood investigations were within the normal range.

**Diagnosis:**

Coronary artery aneurysm in the left main trigeminal position.

**Interventions:**

The patient was treated with stent-assisted coil embolization.

**Outcomes:**

After complete filling of the aneurysm with coil, the microcatheter was withdrawn and the stent released in the descending branch. Two stents were successfully implanted.

**Lessons:**

There is no clear consensus on the optimal therapy for patients with CAAs. Clinicians should be aware of the possible complications of stent-assisted coil embolization of CAA in the main trunk of the coronary artery.

## 1 Introduction

Coronary artery aneurysms (CAAs) are sometimes incidentally detected by coronary angiography. The most serious complications of CAAs include rupture and ischemia due to embolism or thrombosis, especially when associated with significant coronary artery stenosis. Techniques for endovascular treatment of intracranial aneurysms may be applicable to the management of coronary aneurysms; previous reports have described the use of percutaneous catheter-based treatment using only membrane-covered stents.^[1–3]^ However, the underlying etiology is not well understood, and atherosclerotic disease with pre- or post-stenotic aneurysmal dilatation is often associated with CAAs.^[4]^ Other causes include congenital, vasculitis, endocarditis (mycotic), and vessel wall injury during surgical or catheter/wire manipulation.^[5]^

Most CAAs exhibit slow growth and are typically managed conservatively. However, in rare cases, rapid enlargement or intramural thrombosis may necessitate percutaneous or surgical intervention.^[6,7]^ We herein report a rare case of CAA involving the bifurcation of the left main trunk and the anterior descending and circumflex branches of the coronary artery. We successfully treated the patient using drug-eluting stent (DES) and coil embolization.

## 2 Case report

Our study was approved by affiliated hospital of Hangzhou Normal University ethics committee, and formal patient consent was obtained. A 73-year-old woman presented with a history of paroxysmal episodes of precordial pain since 1 year. The episodes typically lasted for several minutes to half an hour, and were either spontaneously triggered or induced by physical activity. There was no history of dyspnea during the pain episodes. Forty days before admission, she experienced recurrence of chest pain lasting for > 1 hour. The patient was diagnosed at the local hospital with acute non-ST-elevation myocardial infarction with significantly increased troponin I and CK-MB in myocardial zymogram; however, there was no ST segment elevation on electrocardiogram (ECG). The patient was treated with low molecular weight heparin, atorvastatin, metoprolol, aspirin, clopidogrel, and ramipril; however, she continued to experience recurrent episodes of angina after discharge. Subsequently, the patient visited our hospital for further management. She had a history of hypertension (>20 years), hyperlipidemia (>10 years) and was a heavy smoker (20 cigarettes per day for 34 years). There was no history of diabetes or allergy to contrast agents. Her father suffered from myocardial infarction at the age of 30 years.

**Figure 1:**
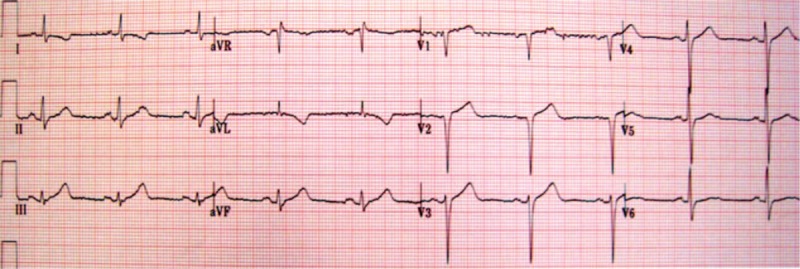
ECG performed during physical examination.

On physical examination, her blood pressure was 162/78 mmHg and her respiratory rate was 21 breaths/min. There were no lung rales or pleural friction. On laboratory investigations, her total cholesterol (TC) was 7.56 mmol/L and low-density lipoprotein cholesterol (LDL-C) was 4.81 mmol/L. Troponin I, CK-MB, creatinine, blood glucose, and other routine blood investigations were normal. The ECG is shown in Figure 1. Echocardiography revealed left ventricular ejection fraction (LVEF) of 55% to 65%, diminished inferior ventricular wall function, asymmetric hypertrophy of the ventricular septum, mild aortic stenosis, and moderate mitral regurgitation. Coronary computed tomography angiography (CTA) revealed an aneurysm (diameter: 9 mm) at the junction of the distal left main coronary artery and the anterior descending branch. The aneurysm was located behind the main pulmonary artery and in front of the left atrial appendage; it was connected with the opening of the circumflex artery (lumen). No obvious lesions were observed in the right coronary artery; however, an eccentric plaque was located between the anterior descending artery and the aneurysm, and had resulted in stenosis.

**Figure 2:**
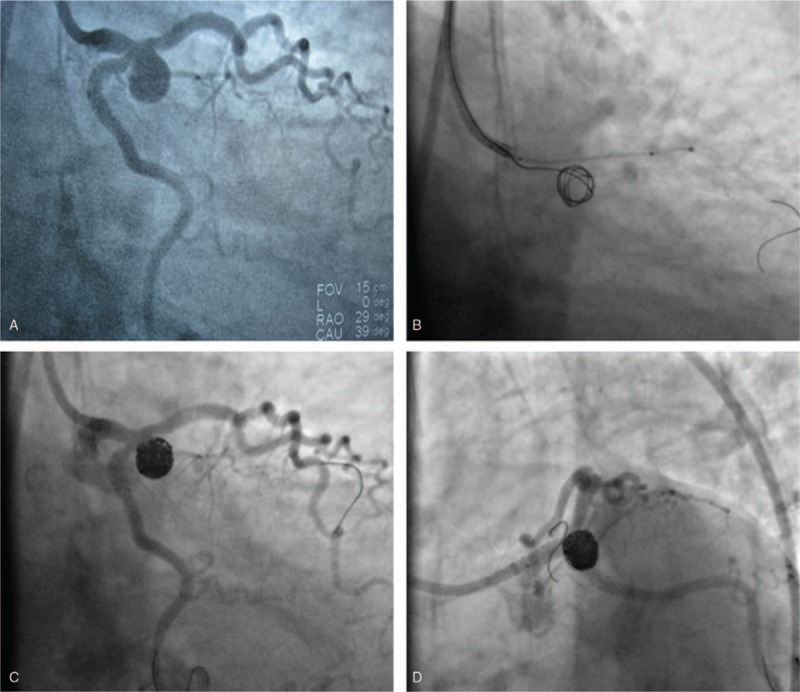
Coronary angiogram of the patient. Coronary artery aneurysm in the left main bifurcation (A); Delivering the coil to the tumor via microcatheter after pre-implantation with a stent in the anterior descending branch (B); aneurysm has adequately filled with coil (C-D).

Coronary angiography revealed significant stenosis of the left main trunk. An aneurysm was found at the bifurcation of the left main trunk and the anterior descending and circumflex branches of the coronary artery. The anterior descending branch was narrowed to 50% to 60% and the circumferential branch opening was reduced to 80% compared with normal branches (Fig. 2A). No obvious stenosis was observed in the right coronary artery or its branches.

On cardiac surgery consultation, the patient was deemed unsuitable for surgical treatment due to the difficulty in accessing the aneurysm area. Therefore, we performed interventional therapy on the patient. Once the guiding catheter was in the right position, a Prowler Plus microcatheter (Terumo Corp., Tokyo, Japan) was navigated to the aneurysm guided by the Agility 14 Soft Tip wire (EV3 Endovascular Inc., Plymouth, MN), and then with the BMW guide wire to the anterior descending branch and the circumflex branch, one each. One DES (Endeavor Resolute 4.0 × 18 mm; Medtronic, Fridley, MN) was implanted between the remote left main trunk and the proximal circumflex branch after balloon dilatation of the proximal circumflex branch. In case of microcatheter damage, 10 atmospheric pressure was released prior to withdrawal of the stent balloon; subsequently, a similar stent was implanted between the remote left main trunk and the proximal anterior descending branch via the stent mesh. Thus, a “fence” was built between the aneurysm and the affected coronary artery. Seven Trufill DCS/Orbit coil systems (8 mm × 10 cm, 7 mm × 10 cm, 6 mm × 8 cm, 5 mm × 8 cm, 4 mm × 6 cm, 3 mm × 4 cm, 2 mm × 2 cm; Codman & Shurtleff, Inc., Freemont, CA) were delivered into the tumor via microcatheter (Fig. 2 B–D), which led to complete filling of the aneurysm. This practice was followed by withdrawal of the microcatheter and release of the stent in the descending branch. Finally, anastomosis of the left main trunk, descending branch and the circumflex branch was performed by routine use of post-expansion balloon. The patient was discharged 4 days after undergoing the procedure and has remained asymptomatic for 3 months. Follow-up coronary angiogram to assess occlusion of the aneurysm and in-stent restenosis would be performed at 1 year.

## 3 Discussion

In the rare case presented here, the CAA was located at the bifurcation of the left main trunk distal and anterior descending and circumflex branches of the coronary artery. CTA scan revealed a relatively large aneurysm (diameter: 9 mm). Routine treatment would be very risky, due to the difficulty of ligation or resection of the aneurysm. Furthermore, any damage to the left main trunk, proximal anterior descending branch, or the proximal circumflex branch could lead to serious consequences. Moreover, it would also be difficult to place the membrane-covered stents in this location, while pharmacological therapy alone would have been inadequate.

Inspired by the treatment of cerebral aneurysm with coil embolization^[8]^ and a case report by Salvatore Saccà et al,^[9]^ we decided to use a stent-assisted coil embolization approach to treat the CAA. The entire procedure was smoothly performed with satisfactory immediate results. The coil filled the aneurysm well, and no rupture or other complications occurred.

CAA is a rare disease. The most common causes are atherosclerosis (52%), congenital malformation (17%), and fungal embolization and arterial dissection (11%).^[10]^ The optimal treatment strategy is still contested,^[11]^ and may involve drug therapy, surgical vascular remodeling (with or without aneurysm ligation or resection), or use of membrane-covered stents. However, none of these approaches was suitable for our patient. Furthermore, no studies have demonstrated remarkable results achieved with drug treatment, while surgical treatment would lead to relatively large trauma. A few reports have described the use of coil embolization for treatment of CAA.^[2,9,10]^ In these cases, although the aneurysms were in the main trunk of the coronary artery, these ones were not located at the bifurcation and one stent was sufficient to seal the aneurysm opening. In our patient, the size and the anatomy of the aneurysm posed a considerable challenge: first, the lesion was located at the trigeminal of the left main trunk, and it is difficult to place protective stent. Second, the aneurysm was huge, and it was necessary to apply a number of spring coils of different sizes to achieve the therapeutic objective of dense embolization. In addition, to prevent rupture of aneurysm by instrumentation and to prevent partial or complete removal of the coil from the stents, both careful manipulation of the microcatheter and precise embolization with an uncontrolled coil were needed. Once a portion of the coil protrudes through the stent mesh into the lumen of the coronary artery, it may induce acute thrombosis with catastrophic consequences. In this patient, excellent therapeutic results were achieved with use of stents combined with multiple coils and meticulous anticoagulation and post-operative follow-up. In summary, we reported a rare case with CAA in the trigeminal position, and successfully treated the patient using drug-eluting stent (DES) and coil embolization. Although the learning curve for this procedure may be relatively long, the efficacy and advantages of this method are worthy of promotion for treatment of aneurysms.

## Author contributions

**Investigation:** Minjie Liu, Kaihan Ren, Mingwei Wang, Fang Zhang.

**Resources:** Yulin Chen, Dongyu Jia.

**Writing – original draft:** Yulin Chen.

**Writing – review & editing:** Yulin Chen.
